# Supplementary material for: Resolving the full spectrum of human genome variation using Linked-Reads
Source: Genome Res. 2019 Apr;29(4):635–45. doi: 10.1101/gr.234443.118 (PMC6442396; doi:10.1101/gr.234443.118)
Supplement: Supplemental Material [file supp_gr.234443.118_Supplemental_longranger-2.2.zip › longranger-2.2.tar.bz2!/lr2.2-public/lib/stan/cmdstan/stan/doc/api/html/functions_vars.html]

Stan: Class Members - Variables


|  |  |
| --- | --- |
|  | Stan  2.5.0  probability, sampling & optimization |


- Main Page
- Related Pages
- Namespaces
- Classes
- Files

- Class List
- Class Index
- Class Hierarchy
- Class Members

- All
- Functions
- Variables
- Typedefs
- Enumerator
- Related Functions

- \_
- a
- b
- c
- d
- e
- f
- g
- h
- i
- k
- l
- m
- n
- o
- p
- q
- r
- s
- t
- u
- v
- w
- x
- y
- z


All Classes Namespaces Files Functions Variables Typedefs Enumerations Enumerator Friends Macros Pages

### - \_ -

- \_alloc
  : stan::math::LDLT\_factor< stan::agrad::var, R, C >
- \_alpha
  : stan::optimization::BFGSMinimizer< FunctorType, QNUpdateType, Scalar, DimAtCompile >
- \_alpha0
  : stan::optimization::BFGSMinimizer< FunctorType, QNUpdateType, Scalar, DimAtCompile >
- \_alphak\_1
  : stan::optimization::BFGSMinimizer< FunctorType, QNUpdateType, Scalar, DimAtCompile >
- \_arguments
  : stan::gm::argument\_parser
  , stan::gm::argument\_probe
- \_bad\_value
  : stan::gm::singleton\_argument< T >
- \_buf
  : stan::optimization::LBFGSUpdate< Scalar, DimAtCompile >
- \_constrained
  : stan::gm::singleton\_argument< T >
- \_conv\_opts
  : stan::optimization::BFGSMinimizer< FunctorType, QNUpdateType, Scalar, DimAtCompile >
- \_cursor
  : stan::gm::list\_argument
- \_default
  : stan::gm::valued\_argument
- \_default\_cursor
  : stan::gm::list\_argument
- \_default\_value
  : stan::gm::singleton\_argument< T >
- \_description
  : stan::gm::argument
- \_fk
  : stan::optimization::BFGSMinimizer< FunctorType, QNUpdateType, Scalar, DimAtCompile >
- \_fk\_1
  : stan::optimization::BFGSMinimizer< FunctorType, QNUpdateType, Scalar, DimAtCompile >
- \_func
  : stan::optimization::BFGSMinimizer< FunctorType, QNUpdateType, Scalar, DimAtCompile >
- \_gammak
  : stan::optimization::LBFGSUpdate< Scalar, DimAtCompile >
- \_gk
  : stan::optimization::BFGSMinimizer< FunctorType, QNUpdateType, Scalar, DimAtCompile >
- \_gk\_1
  : stan::optimization::BFGSMinimizer< FunctorType, QNUpdateType, Scalar, DimAtCompile >
- \_good\_value
  : stan::gm::singleton\_argument< T >
- \_help\_flag
  : stan::gm::argument\_parser
- \_is\_present
  : stan::gm::unvalued\_argument
- \_itNum
  : stan::optimization::BFGSMinimizer< FunctorType, QNUpdateType, Scalar, DimAtCompile >
- \_ldlt
  : stan::agrad::LDLT\_alloc< R, C >
- \_ldltP
  : stan::math::LDLT\_factor< T, R, C >
- \_ls\_opts
  : stan::optimization::BFGSMinimizer< FunctorType, QNUpdateType, Scalar, DimAtCompile >
- \_m
  : stan::prob::welford\_covar\_estimator
  , stan::prob::welford\_var\_estimator
- \_m2
  : stan::prob::welford\_covar\_estimator
  , stan::prob::welford\_var\_estimator
- \_method\_flag
  : stan::gm::argument\_parser
- \_name
  : stan::gm::argument
- \_note
  : stan::optimization::BFGSMinimizer< FunctorType, QNUpdateType, Scalar, DimAtCompile >
- \_num\_samples
  : stan::prob::welford\_covar\_estimator
  , stan::prob::welford\_var\_estimator
- \_pk
  : stan::optimization::BFGSMinimizer< FunctorType, QNUpdateType, Scalar, DimAtCompile >
- \_pk\_1
  : stan::optimization::BFGSMinimizer< FunctorType, QNUpdateType, Scalar, DimAtCompile >
- \_qn
  : stan::optimization::BFGSMinimizer< FunctorType, QNUpdateType, Scalar, DimAtCompile >
- \_subarguments
  : stan::gm::categorical\_argument
- \_validity
  : stan::gm::singleton\_argument< T >
- \_value
  : stan::gm::singleton\_argument< T >
- \_value\_type
  : stan::gm::valued\_argument
- \_values
  : stan::gm::list\_argument
- \_variA
  : stan::agrad::LDLT\_alloc< R, C >
- \_xk
  : stan::optimization::BFGSMinimizer< FunctorType, QNUpdateType, Scalar, DimAtCompile >
- \_xk\_1
  : stan::optimization::BFGSMinimizer< FunctorType, QNUpdateType, Scalar, DimAtCompile >


---

[ Stan Home Page ]

*© 2011–2014,
Stan Development Team.*
